# Vitamin A Requirements in Pregnancy and Lactation

**DOI:** 10.1093/cdn/nzaa142

**Published:** 2020-08-24

**Authors:** Bryan M Gannon, Camille Jones, Saurabh Mehta

**Affiliations:** Division of Nutritional Sciences, and Institute for Nutritional Sciences, Global Health, and Technology (INSiGHT), Cornell University, Ithaca, NY, USA; Division of Nutritional Sciences, and Institute for Nutritional Sciences, Global Health, and Technology (INSiGHT), Cornell University, Ithaca, NY, USA; Division of Nutritional Sciences, and Institute for Nutritional Sciences, Global Health, and Technology (INSiGHT), Cornell University, Ithaca, NY, USA

**Keywords:** breastfeeding, Dietary Reference Intakes, lactation, Nutrient Reference Values, pregnancy, vitamin A

## Abstract

Pregnancy and lactation are critical life stages with unique nutritional requirements, including for vitamin A (VA). Current DRIs for VA were published in 2001. The objective of this review was to identify and categorize evidence related to VA requirements in pregnancy and lactation since these DRIs were formulated. We searched MEDLINE and included articles according to an analytic framework of maternal VA exposure on status and health outcomes in the mother-child dyad. Intermediate and indirect evidence supports that maternal VA intakes can impact the mother's VA status, breastmilk, and health outcomes, as well as the child's VA status and select health outcomes. Food-based approaches can lead to more sustained, sufficient VA status in mothers and children. Research needs include further study linking maternal VA intakes on maternal and child VA status, and further associations with outcomes to determine intake requirements to optimize health.

## Teaser

Evidence synthesis linking maternal vitamin A intakes, maternal and child biomarkers of vitamin A status, and functional and health outcomes since the current Dietary Reference Intakes were released in 2001.

## Introduction

### Vitamin A and nutrient reference values

Vitamin A (VA) is an essential nutrient with critical functions for normal vision, gene expression, growth, immune function, and reproduction. Pregnancy and lactation are critical life stages due to increased requirements to support the growth of the fetus and breastmilk nutrient concentrations (1). Determining nutrient reference values (NRVs) for population groups is required for planning diets, assessing dietary adequacy at both the individual and population level, formulating nutritional education, and providing reference values for nutrition labeling (2).

A general analytical framework to determine NRVs links nutritional intake to clinical outcomes and provides a target to modify this nutrient intake to improve outcomes. This type of data is challenging to obtain and often not present, requiring high-quality longitudinal data linking nutrient intake to clinical outcomes. To fill this gap, the following intermediates are used to generate data and evidence for NRVs: *1*) indicator markers (e.g., biomarkers of nutritional status); *2*) surrogate outcomes (outcomes strongly correlated to clinical outcomes); and in some cases *3*) intermediate markers (outcomes that have not been demonstrated to be strongly linked to clinical outcomes, but have physiological relevance indicating they might be linked to clinical outcomes) (2).

The DRIs are a set of reference values for nutrient intakes of healthy populations targeting the US and Canadian populations. These are comprised of: *1*) the Estimated Average Requirement (EAR): the average daily intake to meet the needs of half of the population; *2*) the RDA: the average daily intake to meet the needs of nearly all (97–98%) of the population; *3*) the Adequate Intake (AI): the average daily intake of a healthy population that is assumed to be adequate when there are insufficient data to determine an EAR; and *4*) the Tolerable Upper Intake Level (UL): the maximum daily intake unlikely to cause adverse health effects (3).

### Current recommendations and methods used in determination of VA requirements in pregnancy and lactation

The current DRIs acknowledge the lack of direct data informing the VA requirement during pregnancy and lactation. These requirements have been estimated by starting with the basal requirements for women and adding additional requirements related to the fetus and breastmilk. Current DRIs are presented in **Table 1**. The requirement for adult women aged ≥19 y is based on the sex-specific calculation for an intake to maintain a minimum acceptable VA reserve to protect from deficiency for 4 mo on a VA-deficient diet (1). This value is extrapolated for females 15–18 y old using metabolic weight. This results in an EAR of 500 and RDA of 700 µg retinol activity equivalents (RAEs)/d for adult women of all ages, and an EAR and RDA of 485 and 700 µg RAEs/d, respectively, for females 15–18 y old.

**TABLE 1:** Summary of 2001 DRIs in pregnancy and lactation (µg RAE/d)^1^

	EAR			RDA			UL		
Age	Women	Pregnancy	Lactation	Women	Pregnancy	Lactation	Women	Pregnancy	Lactation
15–18 y	485	530	885	700	750	1200	2800	2800	2800
19–49 y	500	550	900	700	770	1300	3000	3000	3000

1 EAR, Estimated Average Requirement; RAE, retinol activity equivalent; UL: Tolerable Upper Intake Level.

For VA requirements during pregnancy, the model used takes the base requirement of women and adds the amount estimated to support fetal growth and limited accumulation of VA in fetal liver during gestation. This is estimated to be ∼50 µg RAE/d (1, 4) and is added to EAR and RDA values.

The VA intake and estimated requirements of breastfeeding mothers and their children are intricately linked. For VA requirements during lactation, the model used takes the base requirement of women and adds the average amount of VA delivered through breastmilk. For infants, no functional outcomes of VA status have been demonstrated to respond to VA intake in infants. Currently, an AI is used to reflect the intake of infants primarily fed breastmilk. This is calculated based on average breastmilk intake of 0.78 L/d multiplied by an average milk VA concentration of 485 µg/L. This is rounded to an average of 400 µg RAE/d, which is added to EAR and RDA values. The contribution of milk carotenoids to child VA intakes is not currently included due to lack of data on the bioconversion of these carotenoids to VA in the child (1).

### Overview of VA metabolism

#### General VA metabolism

Dietary VA exists in 2 primary forms, preformed and provitamin A. Vitamin A and provitamin A carotenoids are lipid-soluble, and coconsumption of lipids is required for maximal absorption. Preformed VA is found in animal sources, fortified foods, and supplements predominantly in the form of retinyl esters and has good bioavailability. Provitamin A carotenoids (primarily β-carotene, α-carotene, and β-cryptoxanthin) come primarily from plant-based foods and need to be converted to VA in the body. The bioavailability and bioconversion of provitamin A carotenoids depend on the food matrix and are regulated in a negative feedback loop by VA (5), meaning that estimation of VA equivalency from provitamin A carotenoids is challenging and depends on VA status.

In the intestine, VA and provitamin A carotenoids are incorporated into micelles and absorbed by intestinal enterocytes where provitamin A carotenoids can be cleaved into VA. VA and uncleaved carotenoids are then packaged into chylomicrons and transferred through the lymph into the bloodstream. Chylomicrons can deliver VA directly to target tissues, and subsequently chylomicron remnants are taken up by the liver. As the primary VA storage site, the liver can then store VA as retinyl ester, or complex retinol with retinol-binding protein (RBP) for circulation in the bloodstream. When VA has reached target tissues, it can then be converted to retinal for function in the visual cycle or to retinoic acid, the hormone form of VA that controls expression of numerous genes and drives differentiation of many cell types (6).

#### VA metabolism in pregnancy and placental transfer

There is no de novo fetal VA synthesis; all retinoids needed by the embryo are gained from maternal VA stores. During pregnancy, maternal circulating retinol bound to RBP is delivered to the placenta, where it must dissociate from RBP to cross the placenta and enter fetal circulation. There are several possible mechanisms by which retinol is transferred through the maternal–fetal interface; however, it remains unknown whether free retinol is transferred directly, bound to RBP and transferred via cellular uptake, or transferred through cellular uptake by the stimulated by retinoic acid 6 (STRA6) receptor. It has been shown, however, that RBP itself does not cross this barrier (7, 8). Both retinol-RBP and retinyl esters bound to lipoproteins can provide sufficient retinol equivalents to the embryo to allow for normal embryonic development (9).

In the placenta, VA critically supports embryonic development (10, 11). Retinoids are required for fetal organogenesis, as well as placental maintenance. A 2012 systematic review found that embryonic tissues synthesize retinoic acid after it has been transferred through the placenta (8). Assuming maternal serum homeostasis, fetal VA acquisition remains stable through varying maternal VA intake. As organogenesis develops and the fetus is eventually able to store VA, relative retinoic acid concentrations decrease in the placenta and increase in the embryonic liver. The absence of sufficient VA status results in abnormal development of the heart, which can lead to fetal loss (12), as well as abnormalities in structures derived from the skeletal, respiratory, urogenital, circulatory, and central nervous systems (13–15). In states of excess, high antenatal VA concentrations during embryogenesis and organogenesis can have teratogenic effects (16).

In a recent review, the impact of deficiencies and supplementation of VA and other nutrients on placental function was described. Animal and human studies indicate that deficiencies of numerous nutrients, including VA, can have overlapping proinflammatory effects including elevated concentrations of TNF-α, TNF receptor, and leptin. Supplementation with vitamin D has shown anti-inflammatory effects on the placenta; however, it is unknown whether VA has similar effects due to the overlap in signaling pathways via the retinoid X receptor (17).

#### VA metabolism in lactation

Infants are generally born with low stores of VA, even if the mother has adequate VA status (18). Breastmilk is a rich source of VA, which can provide for the establishment of liver VA stores to support the child in times of low VA intake. Colostrum has substantial concentrations of VA, which decline over time and stabilize through transition and mature milk. Vitamin A concentrations are correlated with milk fat (19).

Exclusive breastfeeding is thought to be able to meet the requirements of the growing child; however, milk retinol and provitamin A carotenoids can vary widely across geographic settings. A multinational study of breastmilk VA and carotenoids found that mean breastmilk retinol ranged across countries from 1.04 to 1.62 µmol/L, and when expressed per lipid ranged from 0.025 to 0.051 nmol/g lipid. Total carotenoids ranged from 0.114 to 0.293 nmol/L, or 2.66 to 8.35 nmol/g lipid, with substantial contributions and geographic variation of provitamin A carotenoids β-carotene, α-carotene, and β-cryptoxanthin (20).

In milk, VA is primarily in the form of retinyl esters associated with milk-fat globule (21, 22). For secretion into milk, retinol is esterified in the mammary gland, which does not appear to follow similar metabolism to the liver, because the mammary gland does not significantly express cellular RBP (cRBP) or lecithin:retinol acyltransferase; instead acyl CoA:retinol acyltransferase might be the primary enzyme (21). Mammary VA can be sourced from either blood retinol or retinyl ester in chylomicrons, which is hypothesized to provide the mechanisms by which breastmilk retinol can reflect dietary VA intake despite minimal changes in blood VA concentrations (23). The uptake of VA to mammary tissue from chylomicrons is closely linked to lipolysis and occurs rapidly (23). Decreases in blood VA and carotenoids during late gestation and early lactation might reflect increased expression of receptors to take up these compounds in the mammary gland for incorporation into colostrum (21).

Although the requirement for VA is increased during lactation, information is limited on the impact that breastfeeding has on maternal VA status and is limited to assuming the increase in VA requirement corresponds to the amount of VA produced in breastmilk (24). Animal studies using swine and rodent models have provided evidence of the transfer of additional dietary VA or supplementation to the mammary gland for incorporation into breastmilk, which translated into improved VA status in the offspring (25, 26).

### Methods to determine VA requirements in pregnancy and lactation

There are numerous types of evidence for determining nutrient requirements for maximizing health outcomes including relating dietary intake to: *1*) direct assessment of nutrient homeostasis or balance; *2*) biomarkers or indicators that adequately reflect nutrient intake or body status; *3*) nutrient-specific functional or physiological outcomes; and *4*) epidemiological relations to conditions of interest.

Assessment of these aspects related to VA is challenging due to the difficulty in accurately estimating total VA intake and the biomarkers and methods to assess VA status. Intake of VA can vary widely within individuals and can be further impacted by seasonality. Further, conversion of provitamin A carotenoids to VA is variable and regulated. VA is lipid-soluble, primarily stored in the liver, and is homeostatically controlled in blood over a wide range of VA statuses meaning that the blood concentration is often not responsive to changes in nutrient intake. For this reason, serum/plasma retinol or RBP have not been used to establish VA requirements (1); however, these biomarkers can have utility indicating the severity of VA deficiency (VAD) as a public health problem at the population level (27).

Additional indicators of VA status include the relative dose–response (RDR) and modified relative dose–response (MRDR) tests that assess the impact of a dose of VA or a VA analog, respectively, to give a categorical estimate of VA deficiency compared with adequacy (28). Retinol isotope dilution (RID) yields a quantitative estimate of total body stores or liver concentrations of VA and responds to dietary VA intake. Longitudinal studies have incorporated paired RID assessments with controlled dietary intake to estimate VA requirements by determining intakes that yield no change in body pool (29). Mathematical modeling of RID kinetics has also been applied to estimate the total VA body pool and provided insights on the absorption, distribution, and flux of VA, as well as conversion of β-carotene to VA (30–32).

## Methods

This review is guided by a specific objective to identify and summarize studies providing evidence toward identifying VA requirements during pregnancy and lactation on maternal and child outcomes since the formulation of the current DRIs. We used a modified analytic framework to guide article inclusion and reporting, and used a literature search and article screening to provide a thorough overview of the available evidence.

### Framework of maternal VA exposure on status and outcome in the mother-child dyad

**FIGURE 1:**
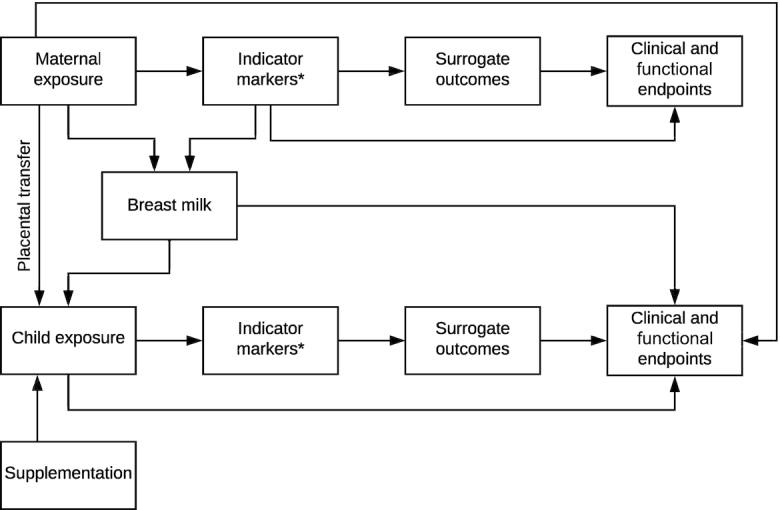
Analytic framework to assess vitamin A requirements in the mother-child dyad. Investigated or biologically plausible associations are represented as arrows between components. Surrogate outcomes are strongly correlated with clinical outcomes, for example, night blindness as a surrogate outcome for xerophthalmia for vitamin A. Changes in indicator markers or surrogate outcomes are expected to reflect changes in clinical outcomes. The analytic framework was used to determine article relevance to the review objective and also categorize synthesis of results from included articles. *Indicator markers include biomarkers reflecting deficiency, adequacy, or excess nutrient status, for vitamin A corresponding to serum/plasma retinol or retinol-binding protein, relative dose–response, modified relative dose–response, and total body stores of vitamin A. Breastmilk is used both as an indicator marker for women and also as the component linking maternal exposure to child exposure during lactation.

We sought to expand the NRV analytical framework (2) to incorporate the paired nature of the mother-infant dyad throughout pregnancy and lactation. The rationale behind this was to allow consideration of studies, data, and indicators that evaluate the relations between maternal VA exposure and both mother and child outcomes. For example, breastmilk VA serves as both a biomarker of maternal intake and status and also directly influences the intake of the child and subsequently child clinical and functional outcomes. Further, some studies report data linking maternal VA intake or exposure to child health outcomes or compare interventions to the mother compared with the child to improve child health outcomes. These putative relations are summarized in the analytic framework to assess VA requirements in the mother-child dyad (**Figure 1**), which was used to determine article relevance to the review objective and also categorize synthesis of results from included articles.

### Search strategy and methodology

In order to target research published since the formulation of the current VA DRIs, MEDLINE was searched via PubMed from January 1, 2000, through December 28, 2019, using the following search string: (“vitamin A”[tiab] OR “vitamin A”[mesh] OR retinol[tiab] OR retinyl[tiab]) AND (pregnancy[mesh] OR pregnancy[tiab] OR pregnant[tiab] OR lactation[mesh] OR lactation[tiab] OR lactating[tiab] OR “breast feeding”[mesh] OR “breast feeding”[tiab] OR “breast fed”[tiab] OR “breast feed”[tiab] OR placenta[mesh] OR placenta[tiab] OR placental[tiab]). Articles were screened by title and abstract for relevance to the review objectives in human or other animal studies. Relevant results were categorized in a narrative style according to the analytic framework (Figure 1). Reviews and meta-analyses were prioritized for reporting if available; all primary studies were not individually listed due to the broad scope of this review.

## Results

### VA population status estimates during pregnancy and lactation

Ensuring adequate VA status remains a challenge for public health globally. Early estimate of the global prevalence of VAD in pregnant women for the year 2000 was that 19.8 million pregnant women had inadequate VA status, of which 7.2 million were VA deficient, and 6.2 million pregnant women experienced night blindness during gestation (33). This corresponds to a global prevalence of 18.4% for inadequate VA using serum or breastmilk VA <1.05 µmol/L, and 5.8% using gestational night blindness (33). Updated estimates from the WHO reflecting data from 1995–2005 indicated 19.1 million pregnant women with VAD (34).

### VA intakes during pregnancy and lactation

Numerous studies have demonstrated a substantial prevalence of inadequate VA intake or status of women of reproductive age, pregnant, and lactating women. A vast majority (∼80%) of women of reproductive age in the United States were not meeting nutrient intake guidelines according to the NHANES 2003–2008. Further, there were differences in intake between ethnic groups and socioeconomic strata (35). A cross-sectional analysis was performed of a nationally representative sample of 1003 pregnant US women aged 20 to 40 y from the 2001–2014 NHANES, which revealed that 15% of pregnant women were not consuming the EAR for VA, even with the use of dietary supplements (36).

Pregnant women receiving obstetric care in the United States received vitamin supplementation with 1500 µg RAE/d (50% as β-carotene); however, hypovitaminosis A was prevalent in 17%, 18%, and 23% of women during the first, second, and third trimester, respectively (37). Reported prevalence of inadequate intakes in pregnant women in other similar studies was determined to be 40% (38) and 74% (39) in Indonesia, and 10% in Canada (40). In Scotland, 69% of pregnant women were consuming <700 µg RAE/d and 25% were consuming <350 µg RAE/d (41).

In lactating women, prevalence of inadequate intake was 57% in Indonesia (42) and 58% in Brazil (43). A high prevalence of VAD was detected by the MRDR test in 6-mo-old breastfed infants in Senegal (44).

An investigation of the effect of antenatal dietary intake of antioxidant vitamins on maternal plasma concentrations showed that mean ± SD total VA intake from food and supplements was 656 ± 1062 µg RAE/d, giving evidence that plasma retinol was less than the reference nutrient intake from early pregnancy to delivery (41). Maternal VA intake was associated with maternal plasma retinol in early pregnancy, but was not associated with plasma retinol at delivery. In contrast, maternal β-carotene intakes were correlated with maternal plasma β-carotene concentrations in early pregnancy and at delivery (41).

In pregnant women in Indonesia, a 2 × 2 factorial design delivered 8.4 µmol VA, 1.07 mmol iron, both nutrients, or double placebo daily for 8 wk. The MRDR test indicated that VA status was significantly improved with the administration of VA supplementation (VAS), and more so when VAS was combined with iron supplementation (45).

Although the risk of inadequate VA intake is prevalent globally, there is also a potential double-burden of VA malnutrition as reported in pregnant and lactating women in Guatemala (46). Prevalence of inadequate intakes were 31% in rural and 9% in urban areas. Simultaneously, 21% of urban women had intakes >1500 µg RAE/d; we note that this cutoff is lower than the UL; however, the study raises the concern of potentially elevated VA intakes with high consumption of fortified foods.

### Maternal VA intake and maternal outcomes

#### Evidence for maternal VA intake on maternal VA status

A 2019 narrative review summarized VA metabolism and epidemiology during the pregnancy period (16). To avoid potential teratogenic effects of VA excess, maternal serum retinol concentrations decrease toward the end of pregnancy and fetal hepatic reserves by the time of birth are low. There is also an overall increase in maternal blood volume and accelerated fetal development, thus the risk of maternal VAD is subsequently increased. The value of this assessment has been questioned, because the gestational process is itself inflammatory (16), and the decreases in serum retinol that accompany inflammation (47) can cause an underestimation of dietary VA status during the end stages of pregnancy.

#### Evidence for maternal VA intake on maternal postpartum indicators

Postnatal VA intakes or supplementation have been investigated for associations with blood-based biomarkers of maternal VA status or breastmilk retinol concentrations. The 2016 Cochrane systematic review of VAS postpartum concluded that there was no significant protective effect of VAS on maternal or infant morbidity or mortality (48), despite increased breastmilk retinol concentrations.

##### Blood-based indicators

A hospital-based observational study in Brazil quantified serum retinol concentrations over the course of lactation and assessed correlations with reported maternal dietary intake. VA intake was significantly associated with maternal serum retinol (*r*  =  0.403) (43).

Higher VA intake or supplementation has been associated with higher maternal serum retinol concentrations (43, 49), and immediate postnatal VAS was shown to increase maternal VA stores (50). In lactating Indonesian women, VA status, as measured by the MRDR test, throughout the 3 consecutive months of early lactation showed a constant decrease in VA status. Thereafter, daily supplementation of 8.4 µmol VA for 35 d resulted in increased VA status (49). Antenatal VAS has also been associated with an increased ratio of mitogen-induced proinflammatory:anti-inflammatory cytokines, indicating that by lessening VAD, VAS might strengthen maternal immunity to cellular infections (51).

In lactating women in Ghana, 2 levels of high-dose VA demonstrated increased VA stores measured by the MRDR assay at 1, 3, and 5 mo postdosing; however, serum retinol concentrations were not different following VAS (52).

##### Breastmilk retinol: animal studies

A study in rats concluded that chronic insufficient VA and zinc intake might alter tissue retinol metabolism and breastmilk retinol concentrations without decreasing maternal free or RBP-bound retinol during lactation. In a 2 × 2 factorial design, rats were fed diets marginal in either VA, zinc, both nutrients, or a control diet from preconception through lactation. All nutrient-deficient rats had higher plasma retinol, lower circulating RBP, and lower hepatic cellular RBP than the control group; however, the mammary gland expression of RBP and cRBP were not significantly affected by the chronic marginally deficient VA diet (53).

Rats were fed high and low amounts of VA throughout pregnancy and early lactation, and higher VA intakes resulted in 2.5-fold elevated milk VA concentrations. This was paralleled by elevated dam and pup liver VA, as well as dam mammary tissue VA; however, dam plasma VA was not different between groups (54). The increased mammary VA was maintained for ≥7 wk after the end of the lactation period (55). Similarly, calves fed colostrum from cows supplemented with VA during late gestation had higher plasma retinol for 14–30 d after birth, compared with calves fed colostrum from unsupplemented cows (56).

Sows fed provitamin A maize throughout gestation and lactation had higher milk VA than sows that received a high-dose VAS at the beginning of gestation and consumed a low-VA diet (1.36 ± 1.30 compared with 0.93 ± 1.03 µmol/L, respectively). Sow livers at the end of the study had similar VA concentrations, indicating that constant dietary VA led to higher VA in milk despite similar sow VA status (57).

In a swine-piglet lactation model, a dose of α-retinol, an isomer of VA that does not bind RBP, was given to sows to trace chylomicron delivery of VA. The α-retinol dose was rapidly taken up into milk, peaking at 7.5 h postdosing, consistent with chylomicron delivery. The majority (62%) of the dose ended up in the sow liver, whereas a substantial portion (15–26%) ended up in the liver of nursing piglets (58).

##### Breastmilk retinol: human studies

Several studies have provided evidence of antenatal VAS increasing breastmilk retinol (59, 60). Maternal dietary micronutrient intakes were related to breastmilk concentrations for breastfeeding mothers in Indonesia using 3-d weighed diet records. The median (quartile 1, quartile 3) daily intake of mothers was 501 (319, 841) µg RAE whereas the VA concentration in breast milk was 13.3 (9.7, 18.6) µg RAE/g fat. For each unit increase (milligram) of VA intake, breastmilk retinol was significantly increased by ∼24% in both unadjusted and adjusted models (38).

An observational study quantified breastmilk total retinol at 1, 14, and 42 d postpartum representing colostrum, transitional milk, and mature milk, respectively. Retinol concentrations observed were: colostrum (146.9 ± 70.9 µg/100 g), transitional milk (81.8 ± 45.8 µg/100 g ) and mature milk (59.5 ± 51.6 µg/100 g), demonstrating a significant decrease over the course of lactation. No significant correlations between retinol concentrations and maternal dietary intake during lactation (61).

A hospital-based observational study in Brazil quantified breastmilk retinol concentrations over the course of lactation and assessed correlations with reported maternal dietary intake. Breastmilk retinol concentrations were not significantly different by dietary VA consumption. Mean breastmilk VA concentrations were 1.57 µmol/L across collections; the prevalence of milk <1.05 µmol/L was 12%, 14%, and 12% at 3 collection points, respectively. The habitual VA intake during lactation using three 24-h recalls were (mean ± SD): 853 ± 275 µg RAE/d, with 58% consuming less than the EAR for lactation (43).

A cross-sectional study of mother-newborn dyads measured maternal VA intake, blood, and breastmilk. There was no correlation between maternal VA intake and breastmilk VA; however, there were weak significant correlations with VA intake and maternal blood VA (62).

VA concentrations in plasma and colostrum were analyzed in women who gave birth prematurely in Tunisia. The study found that plasma and colostrum VA were positively and significantly correlated (*r* = 0.415). Mean ± SD VA concentrations reported in plasma and colostrum were 51.7 ± 20.0 μg/dL and 57.5 ± 50.1 μg/dL, respectively (63).

Colostrum and serum VA were compared in breastfeeding mothers in Brazil representing high and low income levels. It was found that VA intakes, serum, and colostrum VA were elevated in the high-income level, with use of multivitamins during pregnancy emerging as a significant predictor of these outcomes. For high compared with low income, mean maternal intakes during pregnancy were 872 ± 639 and 1169 ± 695 µg RAE/d, which corresponded to colostrum VA concentrations of 86.7 ± 40.0 and 108 ± 58.6 µg/dL, respectively (64). In a hospital-based randomized controlled trial (RCT) of single-dose oral VAS administered soon after delivery, significantly fewer supplemented mothers, compared with nonsupplemented mothers, had deficient concentrations of breastmilk retinol (<0.7 µmol/L). Subsequently, children of the supplemented mothers experienced significantly less diarrheal episodes, acute respiratory infection, episodes of febrile illness, measles infection, and VAD (65).

A 2002 multicenter RCT of lactating mothers in Ghana, India, and Peru showed that maternal VAS increased breastmilk retinol by 2 mo postpartum (difference in means: 7.1, 95% CI: 3.4–10.8 nmol/g fat), but at 6 and 9 mo postpartum did not have a significant effect (66). Infants of supplemented mothers, by 6 mo of age, were less likely to be VA deficient (serum retinol ≤0.7 µmol/L; 30.4% compared with 37%; 95% CI of difference: −13.7, 0.6%). However, by 9 mo of age, VAS did not make a difference in infant VA status (66). Similarly, an RCT of lipid-based supplements found that the provision of 800 μg RE VA to women from pregnancy through 6 mo postpartum preceded no significant differences in breastmilk retinol between intervention arms, even when women received high-dose VAS (200,000 IU) immediately after childbirth (67).

Combined consumption of single high-dose VAS containing 200,000 IU VA and VA-fortified oil compared with nonfortified oil was evaluated in Morocco. At baseline, the groups did not differ in VA per gram of milk fat. The group consuming fortified oil had higher breastmilk retinol at 3 and 6 mo postpartum, and higher serum retinol at 6 mo postpartum (68).

Another RCT assessed the concentrations of secretory IgA (SIgA) present in colostrum after women were supplemented with retinyl palmitate immediately postpartum. VAS made a significant difference in SIgA concentration (343.9 ± 177.2 mg/dL compared with 501.2 ± 54.5 mg/dL), suggesting that it can modulate maternal antibody production (69).

A study in Bangladesh evaluated VAS (10,000 IU weekly) compared with placebo during pregnancy until 6 mo postpartum on H1N1 vaccine response. Supplementation with VA increased VA concentrations in colostrum by 40.7% (70).

In lactating women in Cameroon, breastmilk VA was compared with dietary intake of other VA biomarkers. Inflammation-adjusted RBP was associated with breastmilk VA in the lowest tertile of intake, but not in the highest tertile. Breastmilk VA per gram fat was negatively associated with inflammatory marker C-reactive protein, but not α-1-acid glycoprotein (71).

##### Breastmilk retinol: systematic reviews

Numerous systematic reviews have been conducted regarding VA intake or supplementation on breastmilk VA and related outcomes. One systematic review analyzed the relation between milk retinol concentrations adjusted for milk fat and unadjusted milk retinol, influences of maternal nutritional factors, and maternal VAS. The retinol:fat ratio and retinol concentrations were highest in colostrum, declined rapidly in early lactation, and stabilized by 2 and 4 wk of lactation, respectively. Retinol and milk fat were positively correlated in mature milk. Milk retinol:fat and retinol were associated with maternal VA intake; plasma VA was only associated with milk VA when intake was inadequate. Supplementation with VA yielded higher retinol:fat and retinol for 3 and 6 mo, respectively (19).

Similar systematic reviews evaluating the effect of VAS in postpartum women came to similar conclusions. Supplementation with VA increased serum and breastmilk VA; however, the effect was limited to the short term and when maternal baseline concentrations were low (72). Another systematic review evaluated high-dose VA on blood and milk VA and found significant elevation in milk VA in 9 of 11 studies and significant elevation in blood in 4 of 9 studies compared with control groups (73). A review of supplementation in Brazilian pregnant and postpartum women found groups receiving VAS had increased breastmilk VA relative to control groups (74).

A systematic review evaluated the relation between maternal blood and colostrum vitamin concentrations. VA (as well as vitamins D, E, and K) did not have significant correlations between blood and colostrum; serum VA was inversely related to colostrum vitamin E. Colostrum VA concentrations were higher than serum, likely indicating active mammary gland transport mechanisms (75).

#### Evidence for maternal exposure or status on maternal outcomes

The need for VA during pregnancy is critical not only for fetal growth and development, but also for health outcomes of the mother. Several animal studies have examined the association of maternal VA intake with maternal health outcomes. A study of pregnant rats, for example, showed that VAD in pregnancy leads to abnormal placental apoptosis. The placenta of rats fed VA-deficient diets throughout gestation exhibited greater neutrophil expression of the cytokine TNF-α and its receptor TNFR1, inducing apoptosis and potentially explaining part of the etiology of VAD in fetal development (76). An investigation in sheep identified a decreased risk in vaginal prolapse in ewes administered vitamins A, D, and E during pregnancy (77). In humans, antenatal VAS has been seen to decrease the risk of coagulopathy, but not other causes of hemorrhage such as uterine atony and surgical or obstetric trauma (78).

##### Pre-eclampsia

Cross-sectional investigations of the adipokine RBP have shown inconsistent associations with the risk of pre-eclampsia (PE). In one evaluation of maternal plasma samples, the median RBP concentration was 8% higher in pregnancies with PE than in normal pregnancies (79); however, a proteomic analysis of maternal serum identified 2.4-fold lower concentrations of circulating RBP in PE patients than in patients with normal pregnancies (80). RBP might serve as a predictor of early-onset PE during the first trimester (81, 82). In the third trimester of pregnancy, PE patients had ∼21% lower maternal serum RBPand ∼23% lower umbilical cord serum RBP compared with women with healthy pregnancies (83).

Other observational studies conclude that the risk of PE could be higher in women with lower VA intake and status. Studies in Brazil and Jordan investigated whether antioxidant nutrient intake was associated with PE using 24-h dietary recall questionnaires. In Brazil, VA intake was low across both PE and non-PE women; however, intake in the group of women who developed PE was significantly lower than in the group of pregnant women who did not develop PE (431 ± 278 µg VA compared with 549 ± 1139 µg VA, respectively) (84). In Jordan, pregnant women with lower intakes of fruits and other β-carotene sources had a higher associated occurrence of PE (85). A study of pregnant women in Canada collected blood at 24–26 wk of pregnancy to quantify midpregnancy antioxidant concentrations in women with early-onset, late-onset, and no PE. Both early- and late-onset PE was associated with combined antioxidants, and independently with maternal lutein concentrations (OR: 0.60; 95% CI: 0.46, 0.77); however, summed carotenoids were not significantly associated in the adjusted models (86). A study of catalase activity in Turkey found lower concentrations of serum retinol in PE women (0.39 ± 0.21 µmol/L) than in both healthy pregnant women (0.57 ± 0.15 µmol/L) and nonpregnant controls (0.74 ± 0.16 µmol/L) (87).

##### Gestational diabetes

Gestational diabetes mellitus can also be predicted by VA, as shown via plasma RBP concentrations (88–90). One review concluded that the presence of gestational diabetes can diminish the effectiveness of antenatal VAS, and that the altered metabolic state can cause increases in VA intake to lead to hepatic toxicity (91). Among diabetic pregnancies, the adipokine RBP does not have a statistically significant association with the occurrence of PE (92). Other studies have investigated the association between higher RBP concentrations and the risk of gestational diabetes, finding both positive (93–96) and negative associations (97).

##### Selected infectious diseases

A clinical trial investigated whether oral antenatal and postnatal VAS modified the protective effect of vaccination against H1N1 infection in mothers and their infants. By 6 mo postpartum, mothers who received VAS had vaccine titer protection 38.7% higher than the nonsupplemented group (70). Pregnant gnotobiotic pigs with either VA-deficient or -sufficient diets were challenged with human rotavirus after having received vaccination, high-dose VAS (100,000 IU), both, or control. Pregnant pigs with VAD had imbalanced innate immune responses and worsened rotavirus infection than VA-sufficient pigs, which the provision of VAS did not resolve (98).

A 2015 systematic review analyzed the effect of antenatal VAS on clinical infection, defined by various investigators as a temperature above 37–38°C during pregnancy or the postnatal period and recorded diagnosis of infectious morbidity. When compared with no VAS, VAS alone reduced clinical infection (RR: 0.45; 95% CI: 0.20, 0.99); however, compared with multiple micronutrient supplementation not containing VA, VAS alone did not have an effect (RR: 0.99; 95% CI: 0.83, 1.18). Multiple micronutrient supplementation that contained VA did not have an effect either (RR: 0.95; 95% CI: 0.80, 1.13), although this finding was based on low-quality evidence (99). An additional systematic review of postnatal VAS found only low-quality evidence on the effect of postnatal VAS on maternal diarrhea, fever, and respiratory infections (48).

In a meta-analysis of clinical trials that provided antenatal and childhood VAS for malaria treatment and prevention, antenatal VAS had no beneficial effect on placental infection (RR: 1.09; 95% CI: 0.95, 1.25), peripheral parasitemia, or new malaria episodes of new clinical malaria (100).

##### Other maternal morbidities

Antenatal VAS was protective against the prevalence (OR: 0.71; 95% CI: 0.52, 0.98) and incidence (RR: 0.58; 95% CI: 0.41, 0.81) of bacterial vaginosis during the third trimester when compared with placebo (101). In observations of maternal morbidities beyond 28 wk of pregnancy, VAS showed a protective effect against symptoms of nausea and faintness (OR: 0.66, 0.75, respectively) as well as night blindness (OR: 0.51), upper gastrointestinal infection (OR: 0.82), and urinary/respiratory tract infection (OR: 0.88) symptoms (102). Among dairy cows, a 100-ng/mL increase in maternal serum retinol during the last week prepartum was associated with a 60% decrease in the risk of clinical mastitis in the first week postpartum (103).

The effect of antenatal VA on maternal anemia has been investigated at length (104–109). A 2012 systematic review observed 17 trials of VA or β-carotene supplementation administered to pregnant women (110) and concluded that there were no effects of supplementation on the risk of maternal anemia (RR: 0.81; 95% CI: 0.69, 0.94), though this finding was also highly heterogeneous (*I*^2 ^= 52%; *P* = 0.04). The 2011 review of maternal and newborn outcomes following antenatal VAS found a protective effect against the risk of maternal anemia (RR: 0.64; 95% CI: 0.43, 0.94), as well as maternal night blindness (RR: 0.70; 95% CI: 0.60, 0.82) and maternal clinical infection (RR: 0.37; 95% CI: 0.18, 0.77) (111). Authors of this review also concluded through meta-analysis that antenatal VAS can reduce the risk of maternal anemia (RR: 0.64; 95% CI: 0.43, 0.94), although in comparing the effect of micronutrients that did or did not include VA, the addition of VA to micronutrients did not improve protection against maternal anemia (RR: 0.86; 95% CI: 0.68, 1.09) (99). Another systematic review found that ferritin was increased in pregnant and lactating women with VAS (112). This was echoed in an additional randomized trial that identified an association between low serum retinol and maternal anemia (<0.70 µmol/L compared with >1.05 µmol/L; OR: 2.45; 95% CI: 1.44, 4.17), identifying VAD as a potential risk factor (113), and a case-control study of anemia in pregnant women, in which low VA led to increased anemia (adjusted OR: 8.38; 95% CI: 1.99, 35.30) (114).

##### Maternal mortality

Evidence to suggest a relation between VAS and maternal health outcomes is currently inconsistent. Cochrane systematic reviews from 2011 and 2012 concluded from meta-analyses that no significant protective effect exists from antenatal VAS against maternal mortality (110, 111). There has not been sufficient evidence of any protective effect of VAS against maternal mortality. A 2015 Cochrane systematic review and meta-analysis of VAS for maternal and newborn outcomes did not find an association between VAS and the risk of maternal mortality (RR: 0.88; 95% CI: 0.65, 1.20) (99). The Cochrane review of postnatal VAS highlights less certain effects of VAS in maternal mortality and suggests that postpartum VAS offers limited, if any, protection against maternal mortality (48).

### Maternal intake on child outcomes

#### Evidence for maternal intake on child VA status

A swine study evaluated single-dose VAS to lactating sows on liver VA storage of their piglets. Two dose levels (1.05 or 2.1 mmol) resulted in elevated piglet hepatic VA concentrations relative to sows receiving no VA; however, there was no difference between the 2 VA dose levels (26). Another study in swine compared sows consuming provitamin A maize throughout gestation and lactation with sows receiving a single high-dose VA supplement at the beginning of gestation on piglet VA status. Sows consuming provitamin A maize had piglets with higher liver VA concentrations despite lower plasma VA concentrations in early life (57).

A rat study fed female rats deficient, marginal, or sufficient diets throughout mating and pregnancy, and half of the rats in each group received a single large VA dose during pregnancy. The VA-deficient group had higher placental:fetal ratios, fewer live births, and lower relative weights for fetal liver, heart, and lung. VA during pregnancy increased relative fetal organ weights but had no effect on number of live births (115).

When their infants were aged 4 mo, mother-infant pairs in Honduras were randomly allocated to continue exclusive breastfeeding or introduce iron-fortified complementary foods. At 6 mo of age, infants did not differ in plasma retinol concentration or prevalence of low plasma retinol; however, this population was identified as having a low prevalence of VAD (116).

The effect of antenatal dietary VA intake on maternal and cord plasma concentrations was investigated. Maternal plasma retinol was correlated with, and slightly higher than, cord blood retinol. Maternal VA intake was not associated with plasma retinol or cord blood at delivery. In contrast, maternal β-carotene intakes were correlated with cord blood β-carotene concentrations (41).

A cross-sectional study of mother-newborn dyads measured maternal VA intake, blood, breastmilk, and cord blood. There was no correlation between maternal VA intake or blood VA concentrations and cord blood VA concentrations; however, there was a weak significant correlation with breastmilk VA concentrations and newborn retinol concentrations (62).

The study in Bangladesh that evaluated VAS (10,000 IU weekly) compared with placebo during pregnancy until 6 mo postpartum found that supplementation with VA increased VA concentrations in cord blood by 21.4% (70).

#### Evidence for maternal intake or indicators on child outcomes

##### Child anthropometry

The prepregnancy and antenatal intake of VA has been shown to be associated with childbirth outcomes, in particular birth weight (117–120). A cross-sectional study in India investigated maternal and cord blood VA along with placental and birth weights. Higher VA in cord blood was associated with higher birth weight, in addition to placental weight and gestational age. Low cord VA concentrations were associated with prematurity and intrauterine growth retardation (121). A cohort study in Poland investigated prepregnancy VA intake and the impact of pollution on birth weight. The negative effect of elevated prenatal particulate matter <2.5 µm on birth weight and length was significant for women below the third tertile of VA intake, but these effects were not significant in women with higher VA intakes (122). An observational study of healthy pregnant women in India identified an association between a VAD antenatal diet and fetal low birth weight. Over 90% of the healthy pregnant women studied had a VA intake less than the RDA. By the end of pregnancy, a VAD diet was significantly associated with fetal low birth weight (OR: 3.78; *P*  =  0.04), and this was exacerbated if the VAD diet extended for the entirety of the pregnancy (OR: 10.00; *P*  =  0.05) (123). Reduced birth weight has also been associated with high antenatal retinol and β-carotene intake (124, 125). In a cohort at risk of macrosomia in Ireland, the association between maternal dietary micronutrient intake and neonatal anthropometry was analyzed. The percentage of women consuming the Irish RDA was 26%, 10%, and 26% for the first, second, and third trimester, respectively. Intake of VA in the third trimester was positively associated with neonatal central adiposity (126).

Excess VA was shown to have detrimental effects in an observational study of fish oil intake in pregnant women. Women who consumed the highest quartile of fish oil intake (≥11 mL/d), and thus 3 times the RDA of VA, gave birth to children with smaller head circumferences and shorter lengths than women who consumed less fish oil during pregnancy (127).

##### Child immune function and infectious diseases

Maternal retinoids play a regulatory role in the fetal formation of immune structures, making maternal VA status a critical component of development. The authors of a 2014 review concluded that postnatal VAS is insufficient to support the infant immune system, and that maternal retinoids regulate the in utero development of secondary lymphoid organs (128). A clinical trial investigated whether oral antenatal and postnatal VAS modified the protective effect of H1N1 vaccinations in mothers and their infants. At birth, cord blood in the supplemented intervention arm was 21.4% higher, and in colostrum 40.7% higher, than in the non-VAS group. Cord blood vaccine titer concentrations did not vary significantly across groups (but, in the placebo arm, did vary by time between vaccination and delivery). By 6 mo of age, there was no difference across groups in infant vaccine protection (70). Evidence for the pleiotropic effects of VA on the fetal immune system was also supported in an animal study of nursing piglets, which found that oral VAS during pregnancy increased maternal IgA and subsequent lactogenic protection and higher offspring survival (74.2% compared with 55.9% survival post porcine epidemic diarrhea virus challenge) (129).

##### Child mortality and morbidity

A number of systematic reviews have evaluated the impact of maternal VAS during pregnancy and lactation on child mortality (120, 130, 131). A Cochrane review evaluating VAS during pregnancy primarily in populations with VAD found a beneficial effect on maternal night blindness, anemia, and clinical infection in women (see Evidence for maternal exposure or status on maternal outcomes above); however, there were no significant effects on fetal or neonatal outcomes including perinatal mortality, neonatal mortality, neonatal anemia, preterm birth, or low birth weight (99).

The 2012 systematic review of trials that administered VA or β-carotene to pregnant women found that supplementation did not have an overall effect on fetal loss, miscarriage, stillbirths, preterm births, or small-for-gestational age, but that it did have protective effects against low birth weight after being administered to HIV-positive pregnant women (RR: 0.79; 95% CI: 0.64, 0.99) (110).

Maternal postpartum supplementation with VA and/or carotenoids has also been reviewed extensively for impact on infant and child mortality. There was no evidence of a reduced risk of infant mortality, neonatal mortality, or morbidities including acute respiratory infections and diarrhea in infants aged ≤6 mo (132, 133). Further, there was a lack of evidence supporting VAS to reduce infant mortality or morbidity between 2 and 12 mo, even though maternal breastmilk VA improved with supplementation (48).

##### Child cognition

Antenatal VAS might not be protective against deficits in cognitive development. A follow-up assessment of child neurodevelopmental function examined the independent and combined effects of antenatal and subsequent newborn VAS on scholastic achievement and cognitive and motor function at 8 y of age. General intelligence, memory, and motor functions were not affected by either antenatal or newborn VAS, though the combined interventions showed significant improvements in scholastic performance (134). In a 10–13-y follow-up of Nepalese children whose mothers received either 7000 µg RE or placebo during the preconceptional through postpartum periods, no differences were found in cognitive or motor development, although more placebo-group children repeated a grade in school (28% compared with 16.7%) (135). Similarly, when compared with combined iron/folic acid supplementation, antenatal VAS led to lower mean scores on child psychometric tests at 7–9 y of age (136). An animal study that exposed Sprague Dawley rats to 2.5 mg/kg all-*trans* retinoic acid in utero found long-term cognitive deficits in the offspring of supplemented mothers (137).

##### Other child health outcomes

Maternal VA status and intake before, during, and after the pregnancy period have been investigated for associations with a wide variety of child health outcomes. A population-based case-control study investigated whether periconceptional intake of VA, alongside several other nutrients, was associated with the eye defects anophthalmia and microphthalmia. No association was evident between higher or lower intakes of VA and the risk of these malformations (138). Prepregnancy nutrition of mothers can modulate the harmful effects of prenatal exposures to pollutants on birth outcomes. Among nonsmoking women with singleton pregnancies, the children of mothers with higher prepregnancy VA intake had higher birth weight (β = 176.05) and less negative effects of environmental toxicant exposures (β = 38.6). Higher prepregnancy VA intake was also associated with lessened effects of pollutant exposure on birth length, compared with lower VA intake (β = −0.3 compared with −1.1, respectively) (122). A 2016 cohort study discovered that serum retinol concentration was negatively associated with child weight, bone mineral content, and bone area at birth (139).

Insufficient maternal VA intake appears to be somewhat associated with the risk of birth defects. A case-control study in Norway evaluated the relation between maternal VA intake and risk of having a baby with a cleft palate only or a cleft lip with or without cleft palate. Comparing the fourth and first quartiles of maternal VA intake resulted in lower odds of cleft palate (OR: 0.47; 95% CI: 0.24, 0.94); however, there was no association between maternal VA intake and cleft lip (140). An observational study of children with and without orofacial clefts in Poland discovered that, although none of the participants were VAD, a higher proportion of mothers of children with orofacial clefts had retinol concentrations above the upper norm for women of reproductive age, compared with mothers of children without orofacial clefts (10.6% compared with 5.8%) (141).

A birth cohort study in Japan showed that in pregnant mothers with a prepregnancy BMI of 18.5–24.9 kg/m^2^, those with low VA intake were more likely to give birth to a child with congenital diaphragmatic hernia (CDH) (OR: 0.5; 95% CI: 0.2, 1.0) (142). Similarly, among normal-weight mothers, CDH was more likely to occur in children born to mothers whose VA intake was <800 µg RAE (OR: 7.2; 95% CI: 1.5, 34.4) (143). Maternal VAD has also been associated with long-term child lung function (144), as well as with decreased incidence of newborn bronchopulmonary dysplasia (145).

A systematic review analyzed the relation between human nutrition during pregnancy and kidney structure and function in offspring. Deficiencies in VA, along with other folate and total energy, were associated with negative outcomes on kidney structure and function using outcomes of kidney volume, proteinuria, glomerular filtration rate, and creatinine clearance (146). Mouse model investigations of embryological development have provided evidence that maternal VAD can inhibit normal fetal pancreatic (147) and anorectal (148) development in utero. In a rat model investigating maternal VAD and perinatal organ growth, neonates born to VA-moderate mothers (rats whose VA concentrations were reduced ∼50%) expressed lower levels of proteins associated with skeletal muscle development, compared with the offspring of VA-sufficient mothers (149).

One review provided evidence from human and other animal studies that decreased VA is related to a variety of biological functions associated with autism spectrum disorder: low VA, and its hormone metabolite retinoic acid, led to decreases in CD38 and resulting decreases in oxytocin. Further, low VA was also linked to hyperserotonemia and decreased melatonergic pathway activity (150).

#### Studies reporting adverse outcomes related to VA during pregnancy

Diets high in VA, particularly if combined with VAS, can result in hypervitaminosis A (151). During pregnancy, excess VA intake can lead to teratogenic effects. Azaïs-Braesco and Pascal (152) thoroughly reviewed evidence related to VA requirements and safety limits during pregnancy. Overall, these teratogenic effects due to hypervitaminosis A are rare; in the 30 y prior, there were <20 reports of teratogenic effects resulting from excess intake of VA. One of the causes of this condition is excess supplementation, which emphasizes the importance of careful consideration for determining VAS requirements, particularly for pregnant and lactating women and their children.

In an early study of rat pregnancy, hypervitaminosis A during gestation resulted in embryonic death, preceded by hydremia, as well as cardiovascular and nervous system defects after 24 h of administration of single-dose VA (800,000 IU/kg) (153). Hypervitaminosis A has also led to altered placental morphology and agenesis (154), as well as fetal congenital malformations (15, 17). Antenatal VAS in animal studies has also been linked to abnormal bleeding (155), amplified early fetal hepatic storage (156), pulmonary oxidative stress (157), and varied birth defects (158).

The risk of teratogenic effects of excess VA intake is increased during the first 6 wk of pregnancy (155). An animal study identified oxidative stress and behavioral change as a result of excess VAS in pregnant and nursing rats (159). In humans, excess VA intake in pregnancy has also been associated with increased risk of birth defects (160). A birth cohort study in Japan found an association between prepregnancy or antenatal VA and β-carotene supplementation and worsened child behavior at 3 y of age (161).

The current UL for VA during pregnancy and lactation is 2800 and 3000 µg RAE for women aged 15–18 y and 19–49 y, respectively (Table 1). This is based on epidemiological evidence of teratogenicity of VA intake during or shortly before pregnancy, where evidence indicates no adverse events at doses of ≤3000 µg RAE/d (1).

#### Studies investigating different strategies for improving child VA outcomes

There are different strategies to improve child VA status and health outcomes, including child VAS and maternal VAS or food-based approaches designed to increase breastmilk VA concentrations and ultimately child intakes. Food-based approaches can comprise dietary diversity and inclusion of VA-rich foods, food fortification, and biofortification increasing provitamin A in staple crops such as maize, sweet potato, or rice (162, 163).

Animal studies have provided highly controlled means to evaluate strategies to increase VA status in children through maternal VA intakes. In a VAD sow/piglet lactation model, a single high VA dose at the beginning of gestation was compared with continuous intake of provitamin A maize feed. Sows consuming provitamin A maize had piglets with higher liver VA, yet lower serum retinol, than piglets from sows receiving high-dose VA (57).

A study in rats compared direct VA and retinoic acid supplementation of the neonate with maternal dietary VA on VA status in the offspring. Direct VA treatment to the neonate provided an immediate increase in tissue concentrations that decreased with time. In contrast, increased maternal VA had a gradual and sustained increase in milk VA as well as pup liver and lung VA concentrations (164). This work agrees with studies of neonatal supplementation in piglets that demonstrated rapid influx and clearance of high-dose VA to target tissues including the lung, spleen, kidney, and adrenals. High-dose VA treatment significantly increased VA concentrations in these tissues but they were reduced to control concentrations by 96 h for lung, spleen, and kidney, and by 240 h for adrenals (165).

Various foods and recipes made from high-VA foods have been evaluated for their potential to contribute to VA intakes in pregnant and lactating women. Weighed intakes of sweet potato breads high in β-carotene were evaluated in Ghanaian lactating women. These breads provided an estimated 1.9 and 3.3 mg β-carotene/d, meeting 12% and 21% of estimated requirements (166). Other VA-rich foods, including red palm oil and small fish, have been analyzed for their potential to contribute to VA intakes in pregnant and lactating women (167, 168).

The impact of fortification strategies on dietary intakes was evaluated in breastfeeding women in South Africa, with comparisons of dietary intakes pre- and postfortification. Before fortification, the mean ± SD intake was 247 ± 318 µg VA, and after this was increased to 462 ± 181 µg VA, although at both time points >90% were reported to be consuming below the EAR for VA. Paradoxically, serum retinol was decreased postfortification from 1.66 to 1.26 µmol/L, although the study authors noted that most women were replete, likely due to postpartum VAS campaigns that were active at the time of the study (169).

##### Public health implications of dietary approaches compared with high-dose supplementation

Primary considerations that relate to public health concerns to achieve optimal VA status include efficacy, safety, cost-effectiveness, and sustainability. Numerous studies have evaluated different approaches, strategies, and techniques to target VA intakes and status in populations, with primary strategies being high-dose supplementation to mothers or children, or food-based approaches including fortification, biofortification, and dietary diversity.

Whereas VAS has been shown to be effective in some circumstances, such as supplementation to children 6–59 mo old for the prevention of child mortality, there are other settings and population groups where data on efficacy are limited and supplementation is not a WHO recommended strategy (170), including during pregnancy and lactation except in settings where maternal deficiency is a severe public health problem to prevent night blindness (171, 172).

Evidence during pregnancy and lactation indicates that food-based approaches that provide continuous sources of VA can lead to more sustained, controlled targeting of VA status in mothers and children and is aligned with known metabolism of VA throughout pregnancy and lactation (173–178). This has been demonstrated in animal studies that have evaluated organ VA concentrations that represent either VA storage in the case of the liver, or target tissues such as the lungs. High-dose supplementation can rapidly target these tissues, but the effects have not been shown to last over the typical interval for high-dose supplementation (e.g., every 4–6 mo). Both fortification and biofortification can be cost-effective strategies to address micronutrient intakes (179, 180), and dietary diversity is typically considered an ideal long-term solution to nutritional well-being.

In addition to multiple modes of delivery of VA interventions, there is a distinction between provitamin A and preformed VA. Preformed VA is well absorbed regardless of dose size, and this VA is stored in the liver for recirculation and can be drawn upon in periods of low intake (6). However, the absorption of preformed VA is not strongly regulated, and excessive, chronic intakes could lead to hypervitaminosis. In contrast, provitamin A needs to be converted to VA in the body, which is under negative feedback induced by VA, and the bioconversion of provitamin A to VA can depend on VA status and food matrix. The variability among bioconversion estimates limits certainty that provitamin A interventions will have the desired impacts on VA status; however, evidence indicates that provitamin A carotenoids are an important dietary contribution to VA status globally (181).

### Special considerations

#### HIV

An observational study in pregnant women living with HIV in Tanzania found a prevalence of 35% with low blood VA concentrations. Correlates of low blood VA included severe anemia and plasma vitamin D, vitamin E, and selenium (182). Observational studies have linked low VA concentrations with increased risk of transmission of HIV from mother to child. However, trials of VAS paradoxically increased the risk of mother-to-child transmission, which could potentially be due to VA increasing HIV replication through direct gene activation or an increase in the receptors essential for HIV binding to lymphocytes (183).

A number of studies have investigated the effects of VAS in pregnant women living with HIV (184–191). In 2009, a meta-analysis of randomized controlled trials of antenatal and postnatal VAS in women living with HIV found that VAS had no effect on maternal morbidity (RR: 0.83; 95% CI: 0.59, 1.17) (192). A 2015 Cochrane systematic review of maternal and newborn health outcomes after VAS in pregnancy concluded that in pregnant women living with HIV, antenatal VAS reduces the risk of maternal anemia and night blindness (99). Two RCTs highlighted in another systematic review (193) provided antenatal VAS to pregnant women living with HIV, and found that VA either had no significant protective effect against maternal mortality (194) or reduced the benefits observed when added to a multivitamin regimen (195).

Several studies have also been published on the effects of antenatal VAS on the health status of children born to women living with HIV (196–201). Heterogeneous evidence has been published regarding the protective effect of antenatal and postnatal VAS on the risk of HIV transmission from mother to child (202, 203); however, after meta-analysis of 4 trials, a 2008 systematic review concluded that VAS does not affect maternal-to-child transmission (RR: 1.06; 95% CI: 0.89, 1.26) (192). In 2 systematic reviews, antenatal VAS has been observed to improve the birth weight of babies born to mothers living with HIV (99, 204). There is no evidence that this VAS had an effect on the risk of preterm birth (RR: 0.88; 95% CI: 0.65, 1.19), of stillbirths (RR: 0.99; 95% CI: 0.68, 1.43), or of under-2 deaths (RR: 1.08; 95% CI: 0.91, 1.29) (192). Supplementation with VA or β-carotene of breastfeeding women living with HIV can increase the concentration of breastmilk retinol (60); however, it also can increase the HIV load in breastmilk (205) and is not recommended.

#### Bariatric surgery

Specific attention has been given to the relation of VA status with both maternal and newborn outcomes for women who have received bariatric surgery. A systematic review of maternal micronutrient status in post–bariatric surgery patients found that ≤90% of them reported VAD during subsequent pregnancy, and that this was associated with both inadequate and excessive gestational weight gain (206). Bariatric surgery does not appear to affect breastmilk VA concentrations (207).

Neonatal health outcomes can also be affected by maternal post–bariatric surgery micronutrient deficiencies. In neonates born of VA-deficient mothers, visual complications, such as bilateral microphthalmia, permanent retinal damage, microcephaly, and hypotonia (208, 209) have been observed. Evidence for these conclusions remain weak (210).

### Carotenoids in pregnancy and lactation

The importance of provitamin A carotenoids, including β-carotene, for meeting VA needs in pregnancy and lactation has been reviewed (211). Trials of β-carotene supplementation were reviewed in 2012 with inconsistent findings. A 2019 supplementation trial of β-carotene in pregnant mice (1 mg/kg weight by oral gavage once every 3 d) discovered that the supplementation disturbed lipid metabolism, inducing glucose intolerance and offering caution in administering β-carotene prenatally (212).

In human trials, maternal β-carotene intakes were correlated with maternal plasma concentrations in early pregnancy, at delivery, and with cord blood β-carotene concentrations (41), as well as in cord blood (213). In a 2016 cohort study that investigated offspring bone mineral content and bone area, maternal β-carotene concentrations were inversely related to neonatal bone mineralization, in spite of direct association of maternal serum retinol with the outcome (139). In a 2005 investigation of antenatal dietary intake of antioxidant vitamins on maternal and cord plasma concentrations, plasma concentrations of VA at birth were slightly lower in cord blood than in mothers, and the association lost significance after adjusting for energy. In contrast, cord blood and maternal β-carotene concentrations were associated (*r* = 0.465) (41).

The carotenoid composition of human milk during the first month postpartum and the effect of β-carotene supplementation from days 4 to 32 postpartum were evaluated in women living in the United States. Breastmilk carotenoid, retinol, and tocopherol concentrations decreased over time, typically reaching concentrations reported for mature milk by day 32. Supplementation with β-carotene did not impact breastmilk β-carotene concentrations, with the authors speculating that transitional milk might be saturated with β-carotene (214).

Prenatal β-carotene supplementation had no significant effect on maternal night blindness or T-cell count in pregnant women living with HIV (8). It was seen, however, to be protective against bacterial vaginosis in the third trimester and 3 mo postpartum, compared with a placebo (prevalence, OR: 0.71; 95% CI: 0.53, 0.96; incidence, RR: 0.65; 95% CI: 0.44, 0.96) (101).

Provision of ∼6 mg β-carotene daily for 60 d to lactating women in Zimbabwe with a supplement of puréed papaya or grated carrots, compared with a placebo, improved women's VA and iron status (215). After intervention, serum retinol increased in all groups and RDR indicated increased VA status in β-carotene and papaya groups, which concurred with greater improvements in hemoglobin in these groups.

A case-control study investigated maternal plasma retinol and carotenoids and evaluated the association with risk of spontaneous preterm birth. Plasma retinol was not associated with spontaneous preterm birth, however above average α- and β-carotene, α- and β-cryptoxanthin, and lycopene were associated with a reduced risk of spontaneous preterm birth (216). Low serum α- and β-carotene concentrations were associated with subsequent development of PE in pregnant women with type 1 diabetes (217).

## Summary

### Updated information on VA requirements in pregnancy and lactation

This review has summarized evidence on the requirements of VA in pregnant and lactating women and their children. Ensuring optimal VA intakes is critical during pregnancy and lactation because this is a critical time of growth and establishment of long-term health, and there are well-established sequelae associated with both deficiency and excess during this time.

Direct determination of requirements of VA in humans is challenging due the relative inaccessibility of the liver as the primary storage site of VA and limitations of available biomarkers of VA status. Despite a lack of direct evidence quantifying maternal VA intake on maternal and child outcomes, intermediate and indirect evidence provides links between maternal VA intake and these outcomes. There is evidence that maternal VA intakes can impact the mother's VA status, breastmilk, and health outcomes, as well as the child's VA status, and select child health outcomes. Dietary approaches that provide continuous sources of VA can lead to more sustained, controlled targeting of VA status in mothers and children.

Current knowledge suggests that the VA intake of pregnant women directly influences the circulating retinol available to be transferred through the maternal–fetal interface, and that the VA made available to the developing fetus is critical for survival, growth, and functioning.

Although some studies yielded mixed results, there is evidence that maternal intake of VA has a significant effect on the breastmilk retinol concentrations available to the infant. Further, maternal VAS is associated with higher concentrations of breastmilk retinol.

For pregnant women living with HIV, antenatal VAS protects against maternal anemia, but does not affect maternal mortality. Across studies of pregnant women, higher VA intake and status are associated with lower risks of PE and clinical infection in mothers. Except in settings where VAD is a severe public health problem, VAS is not recommended to pregnant or lactating women for maternal or newborn outcomes.

### Remaining research gaps

Although numerous studies have provided evidence on the importance of adequate VA status during pregnancy and lactation on maternal and child health outcomes, there remain limited data to quantify average requirements and identify important covariates to personalize intake recommendations. Studies specifically quantifying VA intake during pregnancy and lactation, determining biomarkers of VA status reflecting stores of VA, and evaluating the impact of pregnancy and lactation on VA status are needed to more precisely define requirements during these life stages.

Although VA is critical for fetal growth and development, it is still unknown exactly how retinol is transferred, unbound to RBP, through the maternal–fetal interface (7, 8). Enhanced knowledge on the mechanism of placental retinol transfer could inform future antenatal supplementation efforts. Further understanding of the maternal-fetal VA pathway could help clarify the relation between maternal VA status, circulating RBP, and the risk of PE.

Current evidence remains weak and heterogeneous for the maternal VAD–associated child outcomes of post–bariatric surgery pregnancies. More consistent evidence is needed to determine whether antenatal VAS can reduce the risk of adverse child outcomes under these special considerations.

In the current DRIs, the contribution of breastmilk carotenoids is not considered due to lack of evidence on the bioconversion of carotenoids to VA in the infant. Determining the absorption and conversion in infants will provide a more accurate understanding of the role of provitamin A in meeting VA needs during lactation.

In young children, serum retinol tends to be low, even in children born to mothers of adequate VA status (4). Therefore, further refining of applicable cutoffs in infancy will help improve determination of maternal VA requirements during lactation, and subsequently link to improved child health outcomes.

## Notes

The authors reported no funding received for this study.

Author disclosures: SM has an equity interest in VitaMe Technologies (DBA VitaScan), which is commercializing technology focused on developing point-of-care assays for nutritional status based on his research as a faculty member at Cornell University. CJ is supported by the NIH under award 5 T32 HD087137. BMG, no conflicts of interest.

Abbreviations used: AI, Adequate Intake; CDH, congenital diaphragmatic hernia; EAR, Estimated Average Requirement; MRDR, modified relative dose–response; NRV, nutrient reference value; PE, pre-eclampsia; RAE, retinol activity equivalent; RBP, retinol-binding protein; RCT, randomized controlled trial; RDR, relative dose–response; RID, retinol isotope dilution; SIgA, secretory IgA; UL, Tolerable Upper Intake Level; VA, vitamin A; VAD, vitamin A deficiency; VAS, vitamin A supplementation.
